# Supplementary material for: Autophagy Modulation in Human Thyroid Cancer Cells following Aloperine Treatment
Source: Int J Mol Sci. 2019 Oct 25;20(21):5315. doi: 10.3390/ijms20215315 (PMC6862658; doi:10.3390/ijms20215315)
Supplement: Supplementary file 1 [file ijms-20-05315-s001.pdf]

Supplementary Data 1.

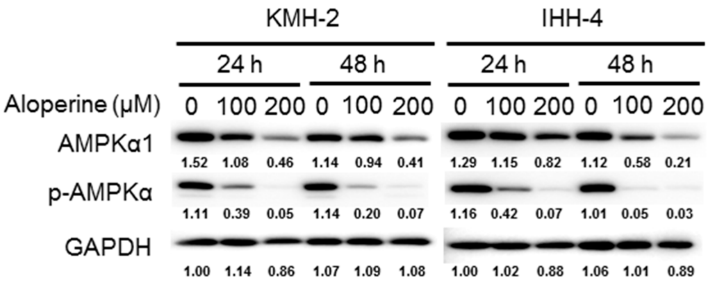

Supplementary Data. AMPK Signaling pathway modulation in human thyroid cancer cells following aloperine treatment. Cells were incubated with aloperine and the expression of AMPK and phospho-AMPK were examined. DMSO was used as a negative control. Numbers under the plots indicate the quantification of protein intensity after normalization with GAPDH. Two independent experiments were performed, and results of one of these are shown.
